# Pre-cueing, the Epistemic Role of Early Vision, and the Cognitive Impenetrability of Early Vision

**DOI:** 10.3389/fpsyg.2017.01156

**Published:** 2017-07-10

**Authors:** Athanassios Raftopoulos

**Affiliations:** Department of Psychology, University of CyprusNicosia, Cyprus

**Keywords:** cognitive penetration, early vision, pre-cueing effects, epistemic role of perception, attention

## Abstract

I have argued (Raftopoulos, 2009, 2014) that early vision is not directly affected by cognition since its processes do not draw on cognition as an informational resource; early vision processes do not operate over cognitive contents, which is the essence of the claim that perception is cognitively penetrated; early vision is cognitively impenetrable. Recently it has been argued that there are cognitive effects that affect early vision, such as the various pre-cueing effects guided by cognitively driven attention, which suggests that early vision is cognitively penetrated. In addition, since the signatures of these effects are found in early vision it seems that early vision is directly affected by cognition since its processes seem to use cognitive information. I defend the cognitive impenetrability of early vision in three steps. First, I discuss the problems the cognitively penetrability of perception causes for the epistemic role of perception in grounding perceptual beliefs. Second, I argue that whether a set of perceptual processes is cognitively penetrated hinges on whether there are cognitive effects that undermine the justificatory role of these processes in grounding empirical beliefs, and I examine the epistemic role of early vision. I argue, third, that the cognitive effects that act through pre-cueing do not undermine this role and, thus, do not render early vision cognitively penetrable. In addition, they do not entail that early vision uses cognitive information.

## Introduction

In previous work (Raftopoulos, 2009, 2014, 2015), I argued that a stage of visual processing, i.e., early vision, is not directly affected by cognition in that its processes do not receive any cognitive feedback in a way that would justify the view that early vision draws on cognition as an informational resource, or, to put it differently, that early vision processes operate over cognitive contents, which is the essence of the claim that perception is cognitively penetrated (see also Pylyshyn, 1999). Early vision, thus, is cognitively impenetrable. This thesis was based on neuroimaging and electrophysiological studies suggesting that cognitively driven attention directly affects perception only at the time scale of late vision that succeeds early vision.

It has been recently argued by philosophers (Cecchi, 2014; Ogilivie and Carruthers, 2015) and cognitive scientists (Vetter and Newen, 2014; Goldstone et al., 2015; Lupyan, 2015) that various pre-cueing attentional effects directly modulate early visual processing itself, in that the signatures of these effects are found within early vision, and since these effects involve cognition, early vision is cognitively penetrated. Fazekas and Nanay (2017) note that if pre-cueing is construed as the expression of cognition driving attention, it would be easy for a defender of the cognitive penetrability of early vision to counteract that pre-cueing is an indirect cognitive effect that, as such, does not entail that early vision is cognitively penetrated. If, however, pre-cueing is seen as the result of the effects of mental imagery on early visual processes, then it entails that early vision is cognitively penetrated.

I defend the cognitive impenetrability of early vision, in three steps. First, I briefly state the thesis that early vision is cognitively impenetrable. In doing so, I explain what is early vision, and I also define in broad terms cognitively penetrability. In the second section, to assess the contention that pre-cueing effects entail the cognitive penetrability of early vision, I examine these effects and argue that they do not affect directly early vision since they so not influence its role in visual processing, to wit, retrieving information from the environment. In the last section, I argue that whether a class of cognitive effects on early vision should be deemed a case of cognitive penetrability hinges on whether these effects affect the epistemic role of early vision since all discussions in the philosophical literature concerning cognitive penetrability are interwoven with the epistemic repercussions of cognitive penetrability.

## Early Vision and Cognitive Penetrability

Early vision includes a feed forward sweep in which signals are transmitted bottom-up. In visual areas (from LGN to IT) the feed forward sweep lasts for about 100 ms. It also includes a stage at which lateral and recurrent processes that are restricted within the visual areas and do not involve cognitive signals occur. Recurrent processing in early visual areas starts at about 80–100 ms and culminates at about 120–150 ms. Lamme (2003) calls it local recurrent processing. The unconscious feed forward sweep extracts high-level information that may lead to categorization and results in some initial feature detection. Local recurrent processing produces further binding and segregation. Studies show that there are early feedback loops, say, from LGN or V1 to MT/V5 and then back to V1, where the recurrent signals engage V1’s neurons to perform different tasks from those performed when V1 received feedforward signals from the LGN (Heinen et al., 2005; Plomp et al., 2015; Drewes et al., 2016).

I said that the feed forward sweep might lead to early categorization. Let me explain this. Familiarity may affect object classification (whether, for example, an image portrays an animal or a face), a process that occurs in short latencies (95–100 ms and 85–95 ms, respectively) (Kirchner and Thorpe, 2006; Liu et al., 2009; Crouzet et al., 2010). Researchers agree that the early classifications in the brain result from the FFS and do not involve cognitive information, nor do they require the activation of object memories. The brain areas involved are low-level visual areas (including the FEF, front eye fields) from V1 to no higher than V4 (Kirchner and Thorpe, 2006), or perhaps a bit more upstream to posterior IT (Peterson, 2003) and lateral occipital complex-LO (Grill-Spector et al., 1998).

The early effects of familiarity may be explained by invoking contextual associations (target-context spatial relationships) that are stored in early sensory areas to form unconscious perceptual memories (Chaumon et al., 2008), which, when activated from incoming signals that bear the same or similar target-context spatial relationships, modify the feed forward sweep of neural activity resulting in the facilitating effects. Thus, what is involved in the phenomenon are certain associations built in the early visual system that once activated speed up the feed forward sweep. This is clearly not a case of top-down cognitive effects on early visual processing.

The early effects may also be explained by appealing to configurations of properties of objects or scenes. Neurophysiological research (Grill-Spector et al., 1998, 2006), psychological research (Peterson, 2003), and computation modeling (Ullman et al., 2002) suggest that implicit associations representing fragments of objects and shapes, or “edge complexes,” as opposed to whole objects and shapes, are stored in early visual areas. The associations that are built, through learning, in early visual circuits reflect the statistical distribution of properties in environmental scenes (Van Rullen and Thorpe, 2001; Delorme et al., 2004). The statistical differences in physical properties of different subsets of images are detected very early by the visual system before any top-down semantic involvement as is evidenced by the elicitation of an early deflection in the differential between animal-target and non-target ERP’s at about 98 ms (in the occipital lobe) and 120 ms (in the frontal lobe). The low-cues could be retrieved very early in the visual system from a scene by analyzing the energy distribution across a set of orientation and spatial frequency-tuned channel (Torralba and Oliva, 2003). This suggests that the rapid image classification relies on low-level or intermediate-level cues (Ullman et al., 2002) that act diagnostically, allowing the visual system to predict the gist of the scene and classify images fast. These cues may be provided by coarse visual information, say by low-level spatial frequency information and the visual system does not have to rely on high-level fully integrated object representations in order to be able to classify rapidly visual scenes.

In Raftopoulos (2009), I argued that early vision processing is cognitively impenetrable because it is not affected directly by cognitively driven attention although attention may affect pre-early vision and post-early vision stages of visual perception. Specifically, cognitively driven spatial attention may determine where one focuses before the presentation of the stimulus and, thus, before the onset of early vision. Or, feature/object based attention may prepare (more about this when I discuss pre-cueing) the perceptual system to process some items in the visual scene faster and more effectively by setting up the values of some parameters of the rules governing the state transformations during perception but the processes themselves of early vision are not affected by attention; attention sets up, as it were, the initial conditions in the transformation equations but the equations themselves are not affected. Finally, attention affects perceptual processing during late vision, which is a post-early vision perceptual stage. This entails that signal transmission during early vision is not affected by top-down signals produced in cognitive areas and is restricted within the visual areas of the brain. Thus, the processes of early vision do not use cognitive information as an information resource and this makes early vision cognitively impenetrable. Pylyshyn (1999) reaches the same conclusion using psychological and behavioral evidence, whereas Raftopoulos (2009) relied on neuropsychological and imaging studies.

The processes of early vision retrieve from the environment the information that will eventually allow perception of a visual scene with as much accuracy as possible. In order to do so, early vision gradually constructs representations of increasing complexity (from variations in light intensities it extracts edges, from edges blobs, from blobs it extracts two-dimensional surfaces, and from these it infers the 21/2 sketch). The representations formed in early vision comprise information about spatio-temporal and surface properties, the shape of the object as viewed by the perceiver, color, texture, orientation, motion, and affordances of objects, in addition to the representations of objects as bounded, solid entities that persist in space and time.

In the discussion thus far I have extensively used the term ‘cognitive penetrability.’ Let me say a few things about what this term means. The term is intended to cover the cognitive influences on perception such that the contents of cognitive states affect the contents of perceptual states through the causal interaction of the cognitive and perceptual states that carry these contents. It is unanimously agreed upon in the relevant literature (the reasons for this are beyond the scope of this paper) that this interaction, in order to signify cognitive penetrability, must be purely mental and should not involve any eye or bodily movements.

For Siegel (2011, 2013, 2016, p. 4), ‘cognitive penetrability’ covers all cases of influences on the contents of experience by prior mental states, including cognitive and emotive states, which causally affect the content of perception such that they influence how things look. Thus, cognitive penetrability occurs when the cognitive effects affect not the selection of the input but perceptual processing itself.

If visual experience is cognitively penetrable, then it is nomologically possible for two subjects (or for the same subject in different counterfactual circumstances, or at different times) to have visual experiences with different contents while seeing *and attending* to the same distal stimuli under the same external conditions, as a result of differences in other cognitive (including affective) states. (Siegel, 2011, p. 5–6).

This is a useful definition of cognitive penetrability because it incorporates the basic desiderata for a conception of cognitive penetrability that is philosophically interesting. It establishes that for cognitively penetrability to occur the same stimulus should being seen. This immediately excludes from being instances of cognitive penetrability various attentional shifts that change the incoming input. In the literature, these cases are unanimously considered not to be cases of cognitively penetrability. It also, much more controversially, excludes any attentional effects from entailing cognitively penetrability because, according to Siegel, they are merely selectional effects that determine the input; the various selection effects where attention selects the input are not cases of cognitively penetrability (Siegel, 2011, 2013, 2016). I think that Siegel is wrong to exclude attention as a potential source of cognitively penetrability since attentional selection effects do occur in late vision and render late vision cognitively penetrated but since I do not have the space to discuss this problem I will simply assume that when cognitively driven attention modulates perceptual processing, this process is cognitively penetrable. This assumption does not affect the main discussion since I argue that attention does not directly affect early vision in any case.

Siegel’s view that CP occurs when cognitive states affect perceptual processing itself if conjoined with the thesis that when they do so the affected perceptual processes operate upon cognitive information, accords with one of Pylyshyn’s (1999) constant themes on cognitive penetrability. This is the thesis that cognition affects perception so that perception and cognition could be deemed to be continuous if cognition causally influences perception directly, that is, if the perceptual processes operate upon the information contained in the affecting cognitive states.

## Early Vision and Pre-Cueing

I have argued (Raftopoulos, 2009, 2014, 2015) that early vision is cognitively impenetrable because it is not directly affected by cognition since the processes of early vision do not use cognitive information, as they do not operate over the contents of any cognitive states. My arguments were based on empirical evidence showing that object/feature based attention and cognitively driven or endogenous spatial attention are delayed and affect the visual areas of the brain (from V1 to IT) after 150 ms post-stimulus, which means that their effects are felt in the visual areas of the brain after the time frame of early vision.

I have also argued that even though the ERP marker of spatial attention P1 is within the time frame of early vision, P1 is in effect the neuronal index of the effects of exogenous, bottom-up spatial attention and, thus, does not signify that early vision is CP. The P1 wave (a component of the ERP waveforms) is larger in amplitude for stimuli presented at the attended location than for stimuli presented at the unattended location. Since the difference is due to the attended location, it is reasonable to assume that the amplitude of the P1 wave is modulated by spatial attention. The effect begins 70–90 ms after stimulus onset, which means that it is clearly an early perceptual and not a post perceptual effect. Spatial selective attention increases the activation of the neural sites tuned to the selected loci. The effect is sensitive to stimulus factors such as contrast and position. It occurs before the identification of the stimuli and is insensitive to the identity of the stimuli. It is independent of the task-relevance of the stimulus, since it is observed for both targets and non-targets. It is also independent of the nature of the task, since it is observed for a variety of tasks ranging from passive viewing to active searching locations. The effect is also insensitive to the cognitive states of the observers (expectations, desires, beliefs, etc.). In that sense, P1 is thought to be an exogenous sensory component elicited by the onset of a stimulus at the attended location.

Recently, philosophers (Cecchi, 2014; Ogilivie and Carruthers, 2015) and cognitive scientists (Vetter and Newen, 2014; Goldstone et al., 2015; Lupyan, 2015) argued against my view that early vision is cognitively impenetrable on the ground that there is empirical evidence suggesting that cognitively driven object/feature-based and spatial attention modulate perceptual processing during early vision. Many studies show that when subjects are instructed to attend to a certain location or attend for a certain object/feature to appear, the neuronal assemblies in the visual brain whose receptive fields are within the attended location, or the neuronal assemblies that encode the feature indicated by these instructions receive a boost in their activation as a result of these instructions and this boost occurs before the appearance of the stimulus. This means that cognitive effects affect perceptual processing from its inception, and, hence, they also affect early vision, rendering it cognitively penetrated.

Cognitive effects are involved in this process because the instructions determine attentional commands (wait for a red letter A to appear, or attend to the upper left part of the screen) to be carried out and these commands require that the subject understand them. When subjects are instructed that a red object will appear on a screen, they use their cognitive resources to understand the instruction and activate their knowledge concerning the color red by activating the neuronal assemblies in the cognitive centers of the brain that store this knowledge. The activation is spread top-down and increases the base-line activation of the neuronal assemblies in the visual areas of the brain that encode the color red. This is a typical example of a cognitively driven attentional effect. Such instructions function as cues directing attention and, since they are given before stimulus presentation, the experimental setting is called pre-cueing. Pre-cueing can occur by cues presented on a screen without any accompanied verbal instructions, as when an arrow ‘up’ appears on a screen. These cues generate attentional commands because the subject understands them and the ensuing attentional effects are cognitively driven.

The problem that pre-cueing effects seem to create for the thesis that early vision is cognitively impenetrable is created by the fact that pre-cueing seems to entail that the processes of early vision are directly affected by cognition in the sense that they operate over some cognitive information. This is a problem because, as we saw, many definitions of cognitive penetrability hinge on whether some perceptual process is directly affected by cognition; should any direct effects on early vision be found, this would entail that early vision is cognitively penetrated.

The cognitive effects on early vision that I discuss are the cases of pre-cueing effectuated by covert shifts of attention. I do not discuss the indirect cognitive effects consisting in shifts of cognitively driven overt attention because these effects are realized through eye- or body-movements and, thus, introduce an external factor in the causal chain by which cognition affects perception, and the existence of such an external factor is almost unanimously thought not to entail cognitive penetrability. Whenever viewers are instructed to attend to a certain location or a certain feature or object to appear, or when they implicitly or explicitly expect some object or feature to appear on a certain location or they expect a specific object or feature to appear on the screen, attention affects perception by modulating the internal on-goings biasing the base-line activation of the neurons that encode the expected stimulus or location. By being internal and not external, this sort of attentional effect is a viable candidate as a cause of cognitive penetrability of early vision.

A word of caution is needed first. I talked about instruction to attend to some location or object/feature, and about expectations that some space will be occupied or that some specific object/feature will appear on the screen and I continued to subsume both attention and expectation effects under the general heading of attentional effects. But expectation and attention are different. When someone expects something, they operate on, or express, information concerning the statistical distributions of objects and spaces in their environment; when expecting O to appear, one attributes an elevated probability to O’s presence in one’s environment. Attention, in contrast, is thought as a mechanism that allows one to focus on, or zoom on what is relevant for one’s purposes. There is empirical evidence showing that the probability of stimulus occurrence and task-relevance are independently manipulated, suggesting that expectations are dissociated from feature-based attention (Kok et al., 2013, 2014). Thus, one should treat the effects of attention and expectation differently. One should note, however, two things. First, even if they are different in nature, their effect on the early visual circuits is the same as we shall shortly see. Second, this dissociation presupposes a conception of attention as some sort of mechanism that acts on information. As I have argued (Raftopoulos, 2009), however, attention is best viewed as the result of the biased competition among pieces of information along the visual circuits. The biases may involve top-down cognitive information and both prior expectations and attentional commands are such biases. If true, this would also explain why they act the same way on visual neurons and it would also allow one to treat them as the same sort of cognitive effect.

Studies of the effects of spatial attention cues presented to a viewer before stimulus presentation show early modulation of perceptual processing (Freiwald and Kanwisher, 2004; Reynolds and Chelazzi, 2004; Carrasco, 2011). Attending to a location may enhance the base-line activation of the neuronal assemblies tuned to the attended location in specialized extrastriate areas V2, V3, V3a, V4, and in parietal regions (Kastner and Ungerleider, 2000; Freiwald and Kanwisher, 2004; Heeger and Ress, 2004; Hopfinger et al., 2004) and in striate cortex V1 (Kastner et al., 1999). This phenomenon refers to the enhancement of the baseline activity of neurons at all levels in the visual cortex that are tuned to a location that is cued and thus this location is attended before the onset of any stimuli. It is called *attentional modulation of spontaneous activity*. The spontaneous firing rates of neurons are increased when attention is shifted toward the location of an upcoming stimulus before its presentation.

This cueing is thought to reflect the effects of the neural processes that occur in response to cues to orient attention to a specific location before the stimulus appears. Spatial attention enhances the sensitivity of the neurons tuned to the attended spatial location by improving the signal-to-noise ratio of the neurons tuned to the attended location over the neurons with receptive fields outside the attended location that contribute only noise. This effect does not determine what viewers perceive in that location because by enhancing the responses of all neurons tuned to the attended location independent of the neurons’ preferred stimuli keeps the differential responses of the neurons’ unaltered and thus does not affect what is perceived. To put it differently, spatial attention determines the focus of the gaze but does not solve the gazing problem of attention. What is perceived depends on the relative activity of appropriate assemblies of neurons that selectively code the features of the stimulus compared to the activity of assemblies that do not code the features of the stimulus and contribute noise. Since the percept depends on the differential response of these assemblies, this effect of spatial attention by not evoking differential responses leaves the percept unchanged; it makes detection of the objects/features in the scene easier but does not determine the percept.

Evidence (Liu et al., 2007; Shibata et al., 2008; Carrasco, 2011; Wyart et al., 2012; Kok et al., 2013, 2014) also suggests that through pre-cueing of object features (instructing a subject to look at a screen for a red object, for example or when a subject expects a particular grating to appear) feature-based attention modulates pre-stimulus activity in the visual cortex. In fMRI experiments designed to examine the effects of feature attention to color and motion on the visual, frontal, and parietal areas, a cue appeared 1 s before the stimulus. The activity within the color sensitive visual areas and the motor sensitive visual areas was increased by attention to color and motion, respectively. This resulted in the relevant visual areas that encode color showing enhanced activation as early as 80 ms after stimulus presentation.

The effects of pre-stimulus feature attention or pre-cueing may act either as a preparatory activity to enhance stimulus-evoked potentials and, thus, the sensitivity to the cued feature, within feature sensitive areas, or they may act to modulate stimulus-locked transients suppressing neural noise. In either case, they make the detection of the target easier, less expensive, and faster. Thus, the preparatory activity that occurs through pre-cues that rely on feature/object based attention increases the base-line firing rate of the neurons preferring the attended stimulus that the participant is instructed to attend to or for which a cue is presented before the presentation of the stimulus. These effects are widespread from V1, V2 to upper levels of perceptual processing. Research suggests [see Montemayor and Haladjian (2015, p. 41–42), and Raftopoulos (2009, Chapter 2), for a list of the relevant research] that the objects in a visual scene are individuated and sometimes are categorized by early vision irrespective of whether they are targets or non-targets or are cued or not, which means that early vision retrieves the required information and individuates all objects in a visual scene, despite the modulation of the pre-stimulus activity due to object/feature-based pre-cueing.

Both effects of pre-cueing reflect a change in background neural activity. These effects are called anticipatory effects and are established prior to viewing the stimulus. In this sense, they do not modulate processing during stimulus viewing but they bias the process before it starts; they do not affect perceptual processing on-line. There are various interpretations of the effects of pre-cueing on the neural activity in the occipital areas of the brain. They may act so as to increase the base-line firing rates of the neurons that encode the pre-cued stimuli; these are cases of gain modulation. Alternatively, they may act so as to suppress noisy neural activity rather than to increase the activity of the neurons that encode the information contained in the pre-cueing signal (Murray et al., 2004; Hegde and Kersten, 2010). It may also be that a variety of mechanisms are available and which one is chosen depends on the task at hand, which means that attention can flexibly solicit different ways to modulate the activity of neurons so as to change visual representations at a cellular level and affect the functional properties of neurons (Gilbert and Li, 2013). In all these cases the net result is the same: anticipatory activity sharpens and optimizes the response properties of the affected neurons according to anticipated stimulus (and this happens independent of whether a stimulus is expected as more likely to appear, or attended to as more relevant to the viewer’s purposes). As such, anticipatory effects do not emerge as part of perceptual competition and in this sense they are not intrinsic to perceptual processing (Nobre et al., 2012, p. 161), which is otherwise unaffected by top-down effects. During the feed forward processing (FFS) and LRP there are no top-down cognitive effects due to pre-cueing, which means that the perceptual processes are data-driven.

What pre-cueing does is to set up the values of some parameters of the transformation rules in feed forward processing. When they set the parameters of the transformation rules, the pre-cueing effects highlight some information in the visual scene, by enhancing the activation of the neurons that encode this information, but they do not create the proximal image or stimulus. What they essentially do is to modulate early perceptual filters; in this sense, they act “as a ‘filter’ that ‘selects’ the information for downstream processing, which may itself be impervious to cognitive influence” (Firestone and Scholl, 2016, p. 23–24). These parameters can be construed as the attentional parameters that weight the effect of sensory signals, as they are postulated in computational models of perceptual attention, such as the model of divisive normalization proposed by Lee and Maunsell (2009). Pre-cueing may increase the value of some parameter and decrease that of another and this results in some input being given priority in terms of subsequent processing but this does not mean that early vision does not retrieve all information in the visual scene.

The pre-cueing effects do not select which information is retrieved from the visual scene once the visual scene has been determined; all information from the visual scene is retrieved in parallel in early vision. In the case of spatial pre-cueing, the anticipatory effects do not determine the percept since pre-cueing enhances responses of all neurons tuned to the attended location independent of the neurons’ preferred stimuli and keeps the differential responses of the neurons’ unaltered. In the case of object/feature pre-cueing, although anticipatory effects enhance the activity of the neurons responding preferentially to the pre-cued object or feature increasing the likelihood that they will be selected eventually for further processing, early vision still retrieves in parallel information concerning all the objects and features present in the visual scene so that these objects be individuated independently of whether they are targets or non-targets.

When attention is used on-line, that is, during visual processing, cognitively driven selective attentional control selects for further processing a specific feature or object in a visual scene by increasing the firing rates of neurons that have a stimulus-evoked response to a particular stimulus; in this case, top-down signals modulate perceptual processing during stimulus viewing. In pre-cueing, processing during stimulus viewing in early vision relies solely on bottom-up processing or top-down and lateral processing restricted within visual areas. This is different from the role of attentional control during visual processing that involves top-down attentional control of the perceptual input.

If pre-cueing does not affect the information retrieved from the visual scene, the relevant cognitive states involved do not affect the selection of the ‘evidence’ or the information against which hypotheses concerning object identity will be tested in late vision. It follows that pre-cueing and the various cognitive effects underlying it do not affect the epistemic role of early vision. As I will explain in the next section, pre-cueing does not entail the cognitive penetrability of early vision.

There is an additional question that needs to be answered. As I have said, in the literature, cognitive penetrability goes hand in hand with the thesis that cognition directly affects early vision in the sense that the processes of early vision use the cognitive contents of the penetrating cognitive states as an informational resource. The question, thus, is the following. Do the pre-cueing effects suggest that cognition affects directly early vision?

Since the cognitive states do not influence the retrieval of information from a visual scene, the cognitive states do not affect perceptual processing itself and, therefore their influence is not direct. This, however, needs arguing for. In view of the fact that the electrophysiological signatures of pre-cueing effects are found within the time frame of early vision, one must examine these electrophysiological signatures. One first response could be that they are carry-over effects of the initial enhanced activation of the relevant feature sensitive areas owing to pre-cueing, that is, of the anticipatory effect of pre-cueing. This means that the fact that they are found during early vision processing does not entail that the contents of the early vision states that participate in these processes are affected by cognitive information, or equivalently, that the processes of early vision operate over such cognitive contents. A way to express this is to say that even though pre-cueing effects set the attentional parameters that we discussed in the previous paragraphs and these parameters in turn affect perceptual processing, the pre-cueing effects act so as to set some initial values but they do not alter the equations that govern the state transformation in which the processing consists. It follows that pre-cueing does not affect the processes of early vision itself, and, thus, does not affect early vision directly.

This conclusion is reinforced by recent studies that examine the role of the FEF in pre-cueing. O’Shea et al. (2004) found early latencies for target/distractor discrimination tasks, as in their study the discrimination by FEF neurons was effective after 100–120 ms after stimulus onset. O’Shea et al. (2004, p. 1063) note that the early latencies discrepancy may be explained by the fact that the repetition of the same target/distractor combination likely resulted in feature priming across the 10 blocks of 80 trials in their experiment and such priming has been shown to produce earlier target discrimination peaks in monkey FEF. It follows that the early onset of target vs. non-target discrimination was likely the result of some sort of feature pre-cueing.

The effects of TMS (Transcranial Magnetic Stimulation) on FEF in relation to pre-cueing was studied by Taylor and Nobre (2007), who applied TMS to the right FEF during the spatial cueing period of a covert attentional task. They found that inducing activity in the right FEF with TMS during the cueing period of a rule-guided covert endogenous attentional orienting task modulated ERPs recorded over visual cortex, which suggests that the TMS applied to FEF altered functional processes related to perception and attention in the visual cortex.

The FEF TMS had a causal impact on visual activity measured with ERPs (Taylor and Nobre, 2007). The earliest effect of TMS was a sustained negative deflection, which became significant after the third TMS pulse, during the interval between the cue and the visual stimulus. This negativity remained until 200 ms after stimulus onset. The data were normalized to a pre-TMS baseline period to emphasize ERP shifts occurring after warning cue onset but before visual stimulus presentation. The normalization shows that this negativity remained present in the ERP until 200 ms after stimulus presentation, which means that this negativity can be interpreted as an effect on visual processing at the time of the attentional modulation of the ERP. In view of the fact that the attentional modulation of the occipital visual areas is delayed in time and occurs after 170 ms post-stimulus, one would expect that the TMS applied to FEF would affect the neuronal activity in early visual areas with a similar time delay, if the TMS effects on FEF affected on line visual processing by controlling top-down attention. Indeed, when Taylor and Nobre (2007) isolated the stimulus-evoked activity by using the peri-stimulus period as the baseline, ERPs differed significantly as a function of FEF TMS starting at 200 ms.

The study by Taylor and Nobre (2007) makes it clear that the TMS is affecting on-going visual cortical activity even prior to visual stimulation, and it is not just affecting the visual cortex’s generation of an ERP. These results mean that

(A) TMS applied to FEF affects neuronal activity in the posterior visual areas prior to the presentation of the stimulus, in accordance with the view that the FEF causally affects modulates the visual activity in posterior visual areas when spatial attention is being allocated.
(B) TMS on FEF continues to affect the visual cortical activity generated by the visual stimulus for about 200 ms after stimulus presentation, which refutes the view that visual stimulation causes the immediate cessation of the cortical processes that were started by the TMS; the pre-stimulus stimulation of FEF keeps playing a role in the control of top-down spatial attention even after stimulus onset;
(C) The effects of the FEF controlled top-down attention are felt on the posterior visual areas at about 200 ms after stimulus onset, which means that their latencies fall within late vision but outside early vision. This last result is very important because it shows that this study does not establish any cognitive effects on early vision but only on late vision.

Another way, (suggested by Gross this volume) is that the attentional parameters in computations provide an example of how cognitive contents can be accessed and operated over without their role in the computation being appropriately inference-like, that is, without there being a logical, reason-giving, relation between the cognitive contents that issue the attentional commands that set the values of the attentional parameters and the contents of the perceptual states that participate in the affected perceptual process. This is important because one of the reasons adduced to support the thesis that early vision is cognitively impenetrable is that cognitive penetrability requires that the cognitive and the perceptual contents stand in a semantic, quasi-logical relation of the sort found in the way the premises of arguments provide reasons for their conclusion. Even though a computational transition might itself be deemed an inference, or inference-like, not all elements of the computation, the attentional parameters, for example, need be quasi-reason-giving. The attentional weights that in Lee and Maunsell’s model are computationally relevant affect computations in a way that does not presuppose that the cognitive contents that set them actually stand in the appropriate reason-giving relation that cognitive penetrability requires.

## Does Pre-Cueing Entail that Early Vision is Cognitively Penetrated?

In the previous section, I examined pre-cueing in detail. The conclusion drawn from that discussion was that pre-cueing does not affect early vision processing itself but acts on it only indirectly since they do not affect the role of early vision, which consists in retrieving information from the environment. The problem now is to decide whether pre-cueing, given its indirect nature, entails that early vision is cognitively penetrated.

To understand better what is at stake with the idea that perception is cognitively penetrable, and decide accordingly whether pre-cueing entails the cognitive penetrability of early vision, one should go back when the discussions about CP of perception started. Hanson (1958), Kuhn (1962), Churchland (1988) and others interpreted findings in psychology and neuropsychology as showing that cognitive states involving propositional/conceptual contents affect perception. This was used as a springboard to mount an attack on the received view in the philosophy of science that there is a theory neutral observational basis on which a rational choice for empirical adequacy between competing theories could be made; the reasonable stance to adopt is that when a theory passes empirical testing it is selected, whereas when a theory fails to pass the empirical tests is rejected in its current form. If perception is cognitively penetrabile, perception becomes theory laden (perception is theory laden if the perceptual processes that produce it are affected by some background theory) and the choice between two alternative and mutually exclusive theories cannot be based on empirical testing. The reason is that since the two theories belong to different paradigms (comprehensive conceptual frameworks) the observations being interpretations made under the influence of the two alternative frameworks differ across paradigms. It follows that there is not a common empirical basis on which the choice between the two theories could be based. From this ensues the incommensurability thesis that bars communication across paradigms; perceptions being modulated by theoretical commitments, the proponents of different paradigms perceive different worlds and assign different meanings to the same terms. This bars communication because there is no neutral basis on which to resolve matters of meaning.

Sellars (1956) sought to undermine one of the tenets of classical empiricism, to wit, the view that one could introspect perception independently of concepts and get to the world, which, thus, is revealed in its own guise without any conceptual influences. This ‘given’ can be used as a neutral basis on which to determine the adequacy of perceptual beliefs. Since the cognitive penetrability of perception undercuts the possibility of such a given, the justificatory role of perception is undermined.

The thread connecting these views is that perception cannot play the epistemological role assigned to it by empiricism because it does not provide a neutral ground on which to decide which of our cognitive schemes is true or false; to the extent that perception is cognitively penetrated, perception’s role in grounding perceptual beliefs is undermined. The cognitive penetrability may affect the epistemic role of perception because it lessens the sensitivity of perception to the data, or because it introduces some sort of irrationality in perceptual processing.

The main motive, therefore, underlying discussions of cognitive penetrability was that cognitive penetrability was thought to undermine the epistemic role of perception in grounding perceptual beliefs, that is, to undermine the extent to which experience could justify some belief. It follows that a cognitive influence on perception is a case of cognitive penetrability if it undermines the epistemic role of perception. However, not all cases of cognitive penetrability undermine the epistemic role of perception and some may even benefit it. One should extend, thus, the definition of cognitive penetrability so that any cognitive influence that affects the epistemic role of perception should be deemed as a case of cognitive penetrability independent of whether it diminishes or enhances this role. This amounts to saying that cognitive influences on perception that do not affect its epistemic role should not be considered cases of cognitive penetrability.

Stokes (2015) argues that cognitive penetrability should be understood in terms of its consequences. This consequentialism captures what is important in all discussions of cognitive penetrability, namely, the consequences of cognitive penetrability for the epistemic role of perception, theory-ladenness of perception, rationality in science, constructivism, etc. According to Stokes, an adequate account of cognitive penetrability should describe a phenomenon (or a class of phenomena) that has implications for the rationality of science, the epistemic role of perception, etc. Stokes (2015) calls this the *consequentialist constraint* on analyses of cognitive penetrability. Stokes proposes *disjunctive consequentialism*, according to which ψ is cognitively penetrated if and only if ψ is a cognitive-perceptual relation that entails consequences for the epistemic role of perception. It should be noted that even though the original considerations, to which Stokes points out, concerning the epistemic impact of cognitive penetrability presupposed that cognitive penetrability undermines the epistemic role of perception and, thus, that it has harmful effects, Stokes (2015, p. 88) notes that on certain occasions cognitive penetrability and the theory-ladenness it induces may be beneficial for perception rather than harmful. It follows that for Stokes, cognitive penetrability occurs when the cognition-perception relation that obtains affects the epistemic role of perception and not when this relation downgrades perception, a view with which I fully agree.

Thus, I concur with Stokes that to determine whether some causal influence on perception counts as cognitive penetrability one should examine the effects of these influences on the epistemic role of perception. I propose, however, that cognitive influences on perception count as cases of cognitive penetrability if they have an epistemic impact on the justificatory role of perception and not only when they undermine the epistemic role of perception.

This sets the following condition for cognitive penetrability, which I call the epistemic criterion for CP.

*Epistemic Criterion for Cognitive Penetrability*: If perception (or a stage of it) is cognitively influenced in a way that renders it unfit to play the role of a neutral epistemological basis, by vitiating its justificatory role in grounding perceptual beliefs, perception (or a stage of it) is cognitively penetrated. If perception (or a stage of it) is cognitively influenced in a way that does not affect its epistemic role in justifying perceptual beliefs, it is cognitively impenetrable.

For the purposes of this paper, I will run this definition in parallel with the more standard definition that we discussed before, namely that a cognitive effect on perception is a case of cognitive penetrability if it affects perceptual processing directly in the sense specified above. In this paper, I will not address in depth the problem of the relations between the two definitions, although the discussion in this paper suggests that the two definitions of cognitive penetrability are inextricably linked. The epistemic criterion for cognitive penetrability entails that to determine whether a perceptual stage is cognitively penetrated, one should examine whether there are cognitive or emotive influences on this stage that affect its epistemic role in grounding perceptual beliefs.

One might argue that the claim that cognitive penetrability occurs only when cognition affects the epistemic role of perception is too strong. Cognitive influences that do not affect the epistemic role of perception are still cognitive influences and, thus, should constitute cases of cognitive penetrability. The discussion in this subsection shows only that there are many types of cognitive penetrability, some of which affect the epistemic role of perception. The objection is on the right track with one caveat. Discussing cognitive penetration, one expects cognition to penetrate perception and one could argue that when it does, it necessarily affects the epistemic role of perception. When, for example, cognitively driven attention indirectly affects perception by selecting the input, this is not a real case of cognitive *penetration*. Be that as it may, one could cogently distinguish between cases of cognitive penetrability that affect the epistemic role of perception and cases of cognitive penetrability that do not. However, I explained above why philosophers take an interest only to those cases in which cognition affects the epistemic role of perception and dismiss the other cases as, philosophically speaking, uninteresting. In keeping with this almost unanimous stance among philosophers, I restrict the term ‘cognitive penetrability’ to those cases in which cognition affects the epistemic role of perception, while recognizing at the same time that from the viewpoint of cognitive science it may make better sense to include in the class of cognitive penetrability all cases in which cognition affects perception independent of the ensuing philosophical repercussions.

Let me say a few things about the impact of cognitive penetrability on the epistemic role of perception. It is intuitive to think that perceptual experience provides defeasible evidence, or warrant, or rational support, or grounds, for endorsing beliefs. It does so directly without any intermediate mental states just because it is perceptual experience. Perceiving p provides prima facie justification, i.e., rational support, for the proposition p. This thesis constitutes the core of the experientialist theories of perceptual justification (Ghijsen, 2016, p. 2). There are many views concerning the way perception justifies perceptual beliefs, which are roughly divided into two main categories; those that fall within internalism and those that fall within externalism. According to internalism, the justification of perceptual beliefs by perception is independent of truth-related factors. Externalists reject this thesis. The two camps differ on the way they interpret and account for the problems that cognitive penetrability engenders for the epistemic role of perception.

Within internalism, the most classical view of perceptual justification is called perceptual or phenomenal conservatism or dogmatism (Markie, 2005; Pryor, 2005; Huemer, 2007; McGrath, 2013a,b; Tucker, 2014), which holds that if it perceptually seems to S that p, then, thereby, S has *prima facie* perceptual justification for the proposition p. Having an experience with content p suffices to give S immediate (meaning that S does not have to believe anything else) *prima facie* justification for p. One of the motives underlying this view is the so-called transparency of perceptual experience; perceptual experience is transparent in that when someone attends to their perceptual experience, they attend to the objects and properties the experience presents to them as in their environment. The phenomenology of the experience presents to them the world as being a certain way. Since perceptual experience presents perceivers worldly states of affairs as in their environment it is rational that they take what their perceptual experience offers at face value and form, prima facie, the belief whose content corresponds to the phenomenal character of their experience.

The problem that cognitive penetrability poses for the epistemic role of perception is that it threatens the role of perception in justifying perceptual beliefs. If prior beliefs affect perceptual processing, this affects the justificatory role of perception. It is arguable that if the belief that X is F causally affects perceptual processing of a visual scene in which an X is present and as a result a viewer has an experience with content “X is F” on which the viewer subsequently bases the belief that X is F, one has a right to suspect that the role of the prior belief in affecting the content of perception undermines the rational support for the perceptual belief; the belief is epistemically compromised. Siegel (2013, p. 702–703) calls the phenomenon of cognitive penetrability leading to epistemically compromised beliefs, the *downgrade principle*.

According to Siegel (2013, p. 707), when the cognitive penetrability of an experience epistemically downgrades the experience by diminishing its justificatory role, this happens because the experience is formed through an irrational process; it is the irrational etiology of the experience that epistemically downgrades it (Siegel, 2013, p. 699–700). The irrational etiology of experience makes it serve as a carrier for forms of influences on beliefs that are epistemically bad. The experiences that are generated through an irrational process, i.e., those that are causally affected by prior mental states in a way that diminishes their justificatory role, generate ill-formed beliefs on account of their etiology.

Not all forms of cognitive penetrability lead to epistemic downgrade. Familiarity, expertise, and perceptual learning in general facilitate rather than hinder the justificatory role of perception (Lyons, 2011; Siegel, 2011, 2013). These are cases in which prior perceptual knowledge changes the way a scene looks, which allegedly is a case of cognitive penetrability, by affecting the features in a visual scene that become salient and, thus, are selected for further processing; expertise and familiarity facilitate pop-out of certain patterns that allow or speed up object recognition. Some forms of CP are beneficial for the viewer in that they increase the viewer’s sensitivity to the visual information in the environment. If cognitive penetrability downgrades experience because of the irrational etiology it introduces, then some forms of cognitive penetrability do not introduce an irrational etiology.

Siegel proposes that to determine whether the influence of a prior mental state on an experience on which another (token) mental state is based epistemically downgrades the experience one should do the following. One should find, first, a belief with the same content as that of the experience. Then, one should find an etiology for this belief that is psychologically similar to the etiology of the experience. If this belief with this specific etiology is doxastically unjustified, the experience has an irrational etiology and has its justificatory role diminished. In other words, one should ask whether the processes leading to the experience, and from there to the belief that is based on this experience, are of the kind that when their corresponding psychological processes that pertain to beliefs are applied to beliefs lead to well-founded or to ill-founded beliefs. As Siegel (2013, p. 717) writes, in view of the fact that it is difficult to define a checkered experience in terms of a sort of cognitive penetrability that is bad, one should rely on one’s sense “of which processes lead to ill-founded beliefs, and of which etiologies of experience are structurally similar to those.”

Lyons (2011, 2015) and Ghijsen (2016), among others, have argued that an inferential, internalistic account cannot explain adequately why cognitive penetrability downgrades perception or why some cases of CP downgrade perception while others do not. I think that the main problem with inferentialism is that it is very hard to defend the ‘Analogy Thesis,’ that is, the view that there is a structural analog between perceptual processes and discursive inferences (Pylyshyn calls them ‘quasi-logical’), that is, the sort of inferences that are involved in drawing inferences from some premises in thought. If the analogy thesis holds, perception is a rational process of belief fixation and the inferences used in perception do not differ from the inferences used in thought, which are called discursive inferences. These inferences are distinguished from ‘inferences’ as understood by vision scientists according to whom any transformation of signals carrying information according to some rule is a form of inference.

It is contestable that there be either in early or late vision discursive inferences (Hatfield, 2009; Raftopoulos, 2011). This undermines the argument that some perceptual processes could be deemed with less rationality on account of their structural affinities to less rational discursive inferences. In this paper, I assume that only taking into consideration externalistic notions, such as the sensitivity of perception to the data, could one hope to achieve an adequate account of the role of cognitive penetrability in downgrading perception.

Siegel, in addition to the thesis that cognitive penetrability downgrades perception because it introduces an irrational etiology, also alludes or explicitly refer to the fact that CP downgrades perception because it diminishes the sensitivity of perception to the environmental data (Siegel, 2013, 2016). Therefore, the fact that in the rest of the paper the analysis of the cognitive effects on early vision hinges on whether they undermine the sensitivity of early vision to the data should not be opposed by Siegel.

Externalists hold the view that to understand the epistemic role of perception in grounding perceptual beliefs, one need invoke truth-related factors, such as the sensitivity of perception to the environmental data and the extent to which perception faithfully reflects the environment. Many externalists are sympathetic to the internalist view that even the person who suffers a cognitively penetrated experience has some *prima facie* reason to hold the corresponding belief. To argue that there is also some reason that the perceiver whose perceptual experience is not subject to cognitive penetrability has better or more evidence to believe the relevant proposition than the perceiver who has fallen prey to cognitive penetrability, the externalists must introduce a more fine-grained account of perceptual evidence that distinguishes between two layers of perceptual evidence and which should be based on truth-related factors.

The first layer must be shared both by the victim of cognitive penetrability and the non-victim, but the non-victim must also possess a second layer of evidence that the victim lacks and which puts the non-victim in a better epistemic position. The first layer of evidence shared by both perceivers must be independent of truth-related factors (since the victim to cognitive penetrability holds a false belief) and must depend only on the phenomenal character of the perceptual experience because this phenomenal content is what the victim and the non-victim share. The first layer of evidence is called phenomenal evidence. The second layer should be sensitive to the fact that the non-victim holds a true belief, while the victim holds a false belief. This sort of evidence is called factive evidence Schellenberg (2013, 2014, 2016a,b). Brogaard (2013) makes roughly the same point by introducing the notion of evidence that grounds the percept, which is the sort of evidence that the perceiver whose perceptual experience is cognitively penetrated lacks, but the perceiver whose perceptual experience is cognitively impenetrable possesses.

For externalists, cognitive penetrability may be epistemically damaging because it creates insensitivity to the distal stimulus, and it may be epistemically beneficial if it increases this sensitivity. The insensitivity to the data can happen in two ways. Either the cognitive states affect perceptual processing whereby information is retrieved from the visual scene and shape the proximal stimulus (the proximal stimulus or image is the iconic information that is retrieved from a visual scene during early vision and is stored in visual circuits) in a way that it ceases to reflect the environment and reflects more one’s conceptual states (Lyons, 2011, p. 301–302). This would be the case if cognition could affect early vision in a way that interfered with this information retrieval.

Or, cognitive penetrability may be epistemically damaging during late vision where hypotheses about the identity of object(s) in the visual scene are formed and tested against the information contained in the proximal image that is transmitted to late vision by early vision (whose output is the input to the processes of late vision) by selecting from the proximal image only confirming information and either ignoring or rejecting disconfirming information. Cognitive penetrability may also speed up object recognition during late vision by selecting faster the relevant information, while ignoring the irrelevant information, which is one of the ways perceptual learning may affect perception.

Wishful seeing, for example, leads through perception to unjustified beliefs because a viewer’s beliefs influence her perception to such an extent that she may see that X is F independent of whether this is true or not. Cognitive penetrability downgrades perception because it makes a viewer’s beliefs insufficiently dependent on her environment and bases them more on her prior mental state; this may make the viewer, simply put, see things that are not there. This is what ‘the insensitivity to the data’ amounts to. “If Jill’s belief that Jack is angry makes her less sensitive to his actual mental state, i.e., less likely to get it right, then this is bad penetration; if it makes her more sensitive, then it’s good” (Lyons, 2011, p. 301–302). Lyons goes on to argue that the insensitivity to the facts undermines the reliability of perception because it increases the probability that the ensuing perceptual belief will be false, and this is related to the details of how cognition affects perception; it is the nature of the penetration and not the penetrator that determines whether cognitive penetrability is bad.

I have argued that the epistemic criterion for cognitive penetrability entails that to determine whether a perceptual stage is cognitively penetrated one should examine whether there are cognitive influences on this stage that affect its epistemic role in grounding perceptual beliefs. To do that, one should delineate first the epistemic role of the perceptual stages.

The epistemic role of perception in grounding perceptual beliefs centers on, but is not exhausted in, the percept because it is the percept that ultimately grounds the perceptual belief whose content matches the content of the percept. The percept that O is F, is formed in late vision because it presupposes that the object and the features in a visual scene have been identified and this takes place in late vision. It follows that the onus of perceptual justification is on late vision; it is late vision that delivers the most important item in the justification process. The details of the processes by which late vision forms the percept have been discussed elsewhere (Raftopoulos, 2011). For the purposes of my arguments here suffices it to say that the epistemic role of late vision is affected by cognitive influences and, thus, late vision is cognitively penetrated.

The epistemic role of early vision is determined by the fact that early vision retrieves from the visual scene information that is fed to late vision and is used for the construction of the percept, in the formation of which the semantic information made available by cognition also plays a crucial role. The iconic information delivered by early vision (the proximal image or stimulus) provides the ‘evidential’ or support basis (should one wish to deny that perception adduces evidence) on which the various hypotheses concerning the identity of objects in the visual scene are formed and tested in late vision. Thus, the role of early vision is to retrieve from the environment the information that will be used by late vision in order for the distal objects in the visual scene to be identified. As I have argued (Raftopoulos, 2009), early vision delivers a structural description of the visual scene that contains information about the 3D shape as viewed from the perceiver, spatio-temporal and surface properties, color, texture, orientation, motion, and affordances of objects, in addition to the representations of objects as bounded, solid entities that persist in space and time.

The problem is to decide whether pre-cueing effects on perception entail that early vision is cognitively penetrable. To do so, one should examine them and determine whether they satisfy the epistemic criterion for cognitive penetrability, that is, whether pre-cueing effects influence the epistemic role of early vision. Since this epistemic role consists in providing late vision with iconic information that is retrieved from the environment, the epistemic role of early vision would be affected by pre-cueing if pre-cueing effects could influence the processes of information retrieval during early vision. If they could, they would affect the sensitivity of early vision to the environmental data and this would render early vision cognitively penetrable.

I claimed above that the pre-cueing effects do not affect the retrieval of information during early vision and, thus, do not influence the proximal image. Thus, the pre-cueing effects do not diminish the sensitivity of early vision to the distal data since all data in the visual scene are retrieved and find their way into the proximal image. This means, in turn, that the pre-cueing does not affect the information that early vision retrieves from a visual scene and is subsequently used in late vision as evidence or the testing ground for the various hypotheses concerning object identity that are formed in late vision. The fact that early vision retrieves from the visual scene all the information that is there, despite the cognitive pre-cueing effects on it, means that the contribution of early vision to the epistemic role of perception is not affected by these cognitive effects and, thus, early vision is not the source of the epistemic downgrade of perception owing to cognitive penetrability. If and when such an etiology emerges, it is due exclusively to the cognitive penetrability of late vision and the way late vision functions. If this is correct, the indirect effects on early vision do not affect the epistemic role of perception; any epistemic effects come from late vision.

## Conclusion

If the cognitive states cannot affect the early visual processes that retrieve information, the information contained in the states of early vision is information retrieved from a visual scene independent of any cognitive influences. It follows that one could not shape the evidence on the basis of which hypotheses concerning the identities of objects will be tested. The information retrieved from the visual scene reflects only the environment and the perceptual makeup of the viewer. In addition, pre-cueing effects do not affect the perceptual processes themselves and, thus, do not entail that early vision uses cognitive information, which means that they do not affect early vision directly; they are indirect effects similar to the shifts of overt attention.

## Author Contributions

The author confirms being the sole contributor of this work and approved it for publication.

## Conflict of Interest Statement

The author declares that the research was conducted in the absence of any commercial or financial relationships that could be construed as a potential conflict of interest.
